# Prevalence of oral lichen planus among a sample of the Egyptian population

**DOI:** 10.4317/jced.51875

**Published:** 2015-02-01

**Authors:** Basma Mostafa, Enji Ahmed

**Affiliations:** 1 Assistant Professor. Oral and Dental Research Division, National Research Centre, Cairo, Egypt; 2 Lecturer. Oral Medicine and Periodontology Dep., Faculty of Oral and Dental Medicine, Cairo University, Egypt

## Abstract

Objectives: To report on the prevalence of oral lichen planus among a sample of the Egyptian population. Study Design: 4470 Egyptian patients, aged 15-75 years, were seen at the outpatient clinic at the Faculty of Oral and Dental Medicine, Cairo University, Egypt. 31.25 % of these patients were males and 68.75% were females. Oral mucosal lesions consistent with oral lichen planus (OLP) were identified both clinically and confirmed histologically (in atypical cases) so that the prevalence of oral lichen planus in this study is 1.43%. Results: 64 patients were diagnosed with OLP (20 males and 44 females). The average age of the affected group was 48.07 years. Associated skin lesions were detected in 15/64 patients (23.44%) and tobacco habits was observed in 20/64 patients (31.25%). The average period of follow-up of the affected cases was 1-2 years, during which two cases developed squamous cell carcinoma of the oral lesion. Conclusions: Within the limitations of this study it revealed the prevalence of OLP among middle-aged women. Atrophic lesions were most frequent, followed by the erosive forms. Anti-HCV circulating antibodies were more common in patients with OLP and, notably, OLP was associated with Diabetes mellitus in 15.63% of patients.

** Key words:**Prevalence, oral lichen planus, Egypt.

## Introduction

Oral lichen planus [OLP] is a chronic inflammatory disease that involves the stratified squamous epithelial tissues. It affects the oral and genital mucous membranes, skin, nails and scalp in addition to esophageal mucosa, larynx and conjunctivae (1).

Several immunological mechanisms of its pathogenesis have been proposed, including antigen-specific cell-mediated immune response, non-specific immunological mechanisms, autoimmune response, and humoral immunity (2,3).

Although the precise etiology of OLP is unknown, in most cases a multifactorial process is considered to be involved, with the participation of genetic, psychological and infectious factors. Some of these factors could act as causal agents, while others may trigger the process (4,5).

The estimated prevalence of the disease in the general population worldwide is between 1–1.5%, with predominance among females in the fifth and sixth decades of life (6). The clinical features of OLP are generally polymorphic and usually consist of bilateral and/or multiple symmetrical lesions, such as white and raised papules or plaques, erosions, or often-painful atrophic lesions (7).

The diagnosis of OLP should be done by clinical and histological examination. However, in classical lesions, it is possible to achieve the diagnosis based solely on clinical appearance. OLP lesions normally last for years with alternating periods of exacerbation and quiescence (4).

Varying prevalence rates of OLP have been reported in different parts of the world, while information regarding the epidemiology and clinical characteristics of this disease in Egypt is incomplete.

The aim of this study is to establish the prevalence of OLP, its clinical characteristics, distribution, and associa-ted findings in a sample of the Egyptian population.

## Subject and Methods

A retrospective and observational study was conducted. The study protocol was approved by the ethical committee at the National Research Center, Cairo, Egypt.

4470 Egyptian patients, aged 15-75 years, were seen at the outpatient clinic at the Faculty of Oral and Dental Medicine, Cairo University, Egypt between January 2012 and April 2014. 31.25 % of these patients were males and 68.75% were females. Oral mucosal lesions consistent with oral lichen planus [OLP] were identified both clinically and confirmed histologically [in atypical cases].

The following clinical data were obtained from the diagnostic charts: gender, age, and clinical presentation of OLP, site affected, presence or absence of symptoms, extraoral manifestations of lichen planus and presence or absence of systemic diseases. Major stressful events [e.g. death of family member, divorce, job loss, major accident] were taken into account. In addition to co-adjuvant local factors such as smoking and oral hygiene [scored as good, regular or deficient] and treatment used to manage the cases were also considered. Records of patients diagnosed with lichenoid lesions were excluded from the sample. In particular, patients with oral lichenoid contact lesions [OLCL] resulting from allergic contact stomatitis [delayed immune mediated hypersensitivity], most commonly in direct relationship to dental restorative materials (8), and also patients with oral lichenoid drug reactions [OLDR], which arise in association with the use of some medications [e.g. oral hypoglycaemic agents and angiotensin-converting enzyme inhibitors] (9).

For the clinical classification and histological discription of OLP, we used the modified World Health Organization [WHO] criteria 2003 (7). Six clinical forms of OLP have been described which are white forms namely reticular, papular, plaque-like and the red forms namely erosive [ulcerated], atrophic [erythematous] and bullous (2).

In the present study, the diagnosis of OLP was generally made based on the clinical aspects of lesions installed in the oral mucosa and sometimes confirmed by the analysis of lesions found on the skin, nails or other mucosa [after referral to the dermatology department at the Faculty of Medicine, Cairo University], if present. A biopsy was only obtained in atypical cases and the material was sent to the Oral Pathology Department at the Faculty of Oral and Dental Medicine, Cairo University for histopathological analysis. The distribution of the oral lesions was recorded for each patient. Incomplete or inaccurate records were not considered. Information was obtained concerning the onset of the oral lesion, associated skin lesions, systemic condition and tobacco habits. Some patients were referred to the Faculty of Medicine, Cairo University for the detection of any undocumented or undetected systemic diseases.

The 2-hours postprandial blood glucose level was measured using SD Check, SD Biosensor, INC, Korea if un-controlled diabetes was suspected on site.

Patients were screened every 3 months and / or when the painful symptoms had become worse. Generally pa-tients with OLP were given topical steroids [Betamethasone 1%] and a surface anesthetic [Lidocaine 1% oral gel] as a treatment, while those with severe painful symptoms [erosive and atrophic forms] medically free patients received systemic corticosteroid therapy.

A descriptive statistical analysis was made using the Statistical Package for the Social Sciences version 12.0 [SPSS Inc., Chicago, USA].

## Results

The final study sample comprised 4470 Egyptian patients aged between 15-75 years [68.75 % females and 31.25 % males] seen in the oral diagnosis clinic at the Faculty of Oral and Dental Medicine, Cairo University, Egypt.

Oral mucosal lesions consistent with lichen planus were identified both clinically and confirmed histologically [in atypical cases] in the 64 patients, 60/64 [93.75] of these presented painful symptoms while in 4/64 [6.25%] patients the lesions were asymptomatic. OLP lesions occurred in 44 females [68.75%] and in 20 males [31.25%] with a female to male ratio [2.2:1]. The prevalence of oral lichen planus in this study was 1.43%. Most of the patients were females between the 4th and 6th decade [n=49/64; 48.45%]. All patients with OLP showed deficient oral hygiene except for two patients who showed regular oral hygiene. These two patients presented with asymptomatic reticular form of OLP on the buccal mucosa and were medically free and non-smokers. The age and sex distribution of the patients are shown in  Table 1. The findings are presented for both sexes combined.

**Table 1:**
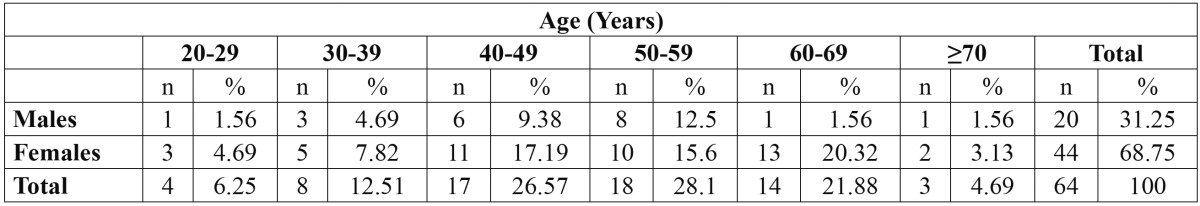
Shows the age and sex distribution of 64 oral lichen planus patients among a sample of the Egyptian population.

**Figure 1:**
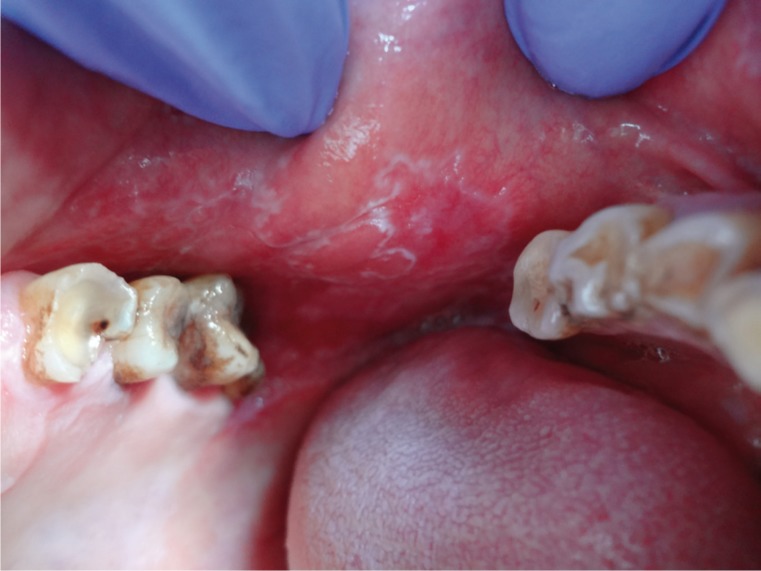
Atrophic LP on the buccal aspect of the check mucosa.

**Figure 2:**
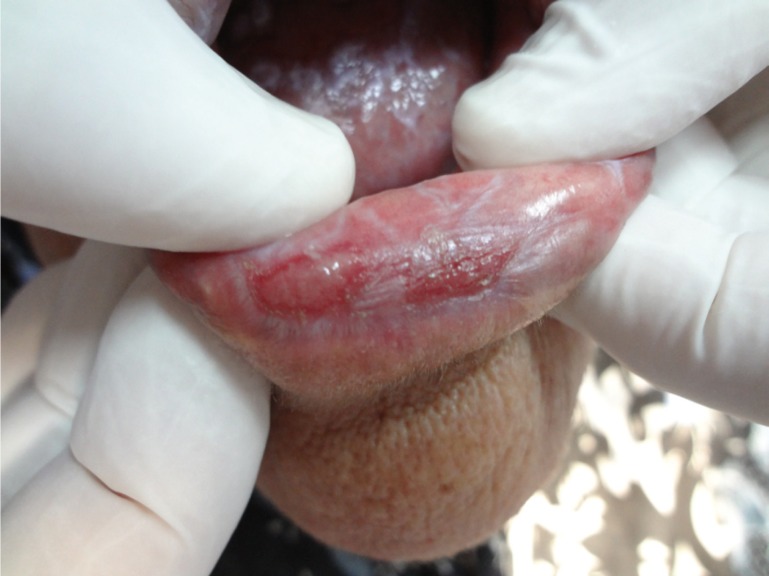
Erosive LP ulcerated lesion on the lower lip.

Six clinical forms of lichen planus were identified: reticular, erosive, atrophic, plaque-like, papular and bullous forms. The red type of OLP [atrophic and erosive lesions] was predominant in this Egyptian sample of patients [59.37% of patients had atrophic OLP as shown in figure 1 meanwhile 20.3% developed erosive type of OLP as presented in figure 2]. Most of the patients studied presented multiple oral lesions. The buccal mucosa was the most affected site [n=52], followed by the gingiva [n=37], lips [n=28], tongue [n= 11] and palate [n=2]. The OLP lesions occurred at different anatomical sites combined. The anatomical distributions of the six clinical forms identified are given in  Table 2.

**Table 2:**
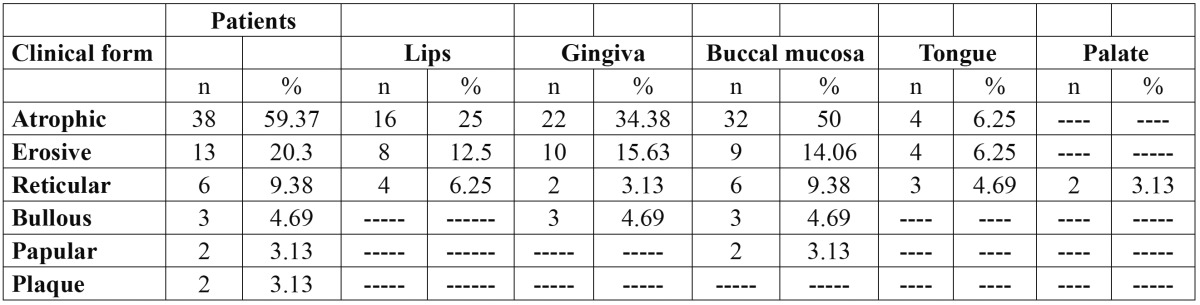
Shows the anatomical distribution of the clinical forms of oral lichen planus among the 64 patients.

Characteristics of the patients with oral lichen planus are presented in  Table 3. Associated skin lesions were detected in 15/64 patients [23.44%] and tobacco habits was observed in 20/64 patients [31.25%].

**Table 3:**
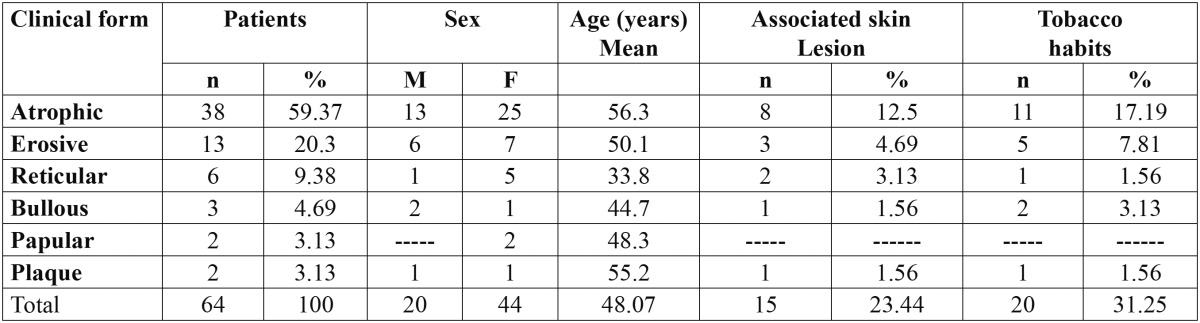
Shows the characteristics of the 64 patients with oral lichen planus among a sample of the Egyptian population at the time of examination.

The incidence of systemic disease among the patients with oral lichen planus is given in  Table 4. Diabetes mellitus was reported in 15.63% [n=10/64] of cases while hypertension was detected in 12.5% [n=8/64]. Viral hepatitis C infection was documented in 11/64 patients [17.9%] and cardiac problems were revealed in 7/64 cases [10.94%]. Chronic renal failure was seen in only 2/64 patients [3.13%]. Psychological stress was considered in 6/64 patients [9.38%].

**Table 4:**
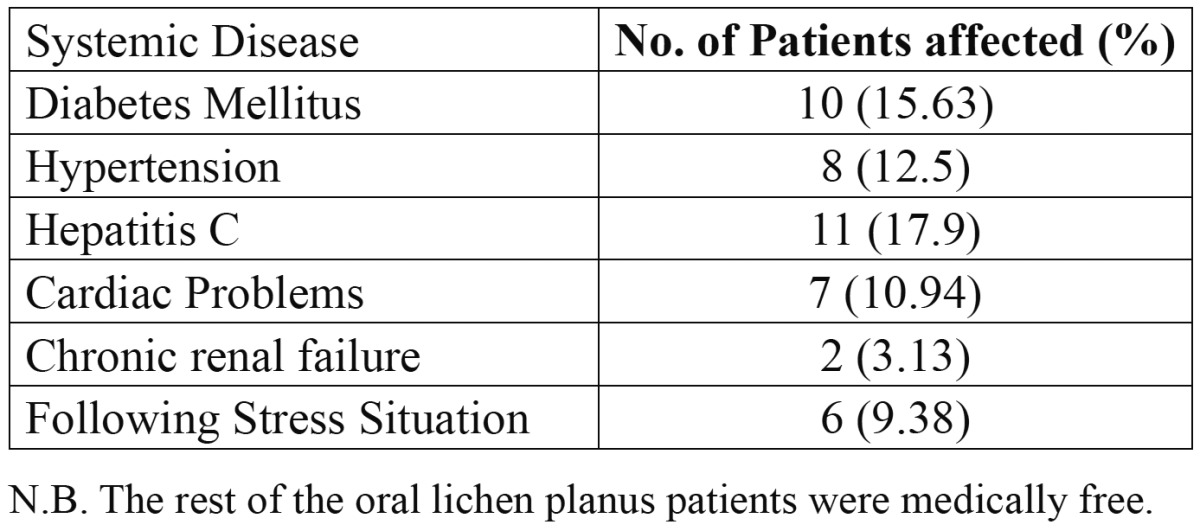
Shows the incidence of systemic disease among 64 patients with oral lichen planus.

All patients showed both remissions and exacerbations of the painful symptoms. Only 2 patients with reticular oral lichen planus did not show up after the first examination and prescription of the topical steroid and surface anesthetic therapies. These 2 patients were systemically free proven by their documented medical data. The remaining patients were followed regularly. The average period of follow-up was from one to two years, during which 2 patients developed oral squamous cell carcinoma at the site of the oral lesion. In this study, 32% of patients reported an exacerbation of OLP symptoms in periods of greater emotional tension and/or anxiety. Burning sensation was the most frequent symptom and was observed in 60% of cases; of these, 76.08% had the erosive form of OLP.

Extraoral manifestations were observed in 33.8% of the patients studied and exclusive oral lesions in 66.2%. According to the histopathological data obtained by analysis of biopsies of atypical cases OLP lesions, basal layer destruction and basal membrane interruption as the result of hydropic degeneration, formation of Civatte bodies, and a juxta-epithelial lymphocyte inflammatory infiltrate with a band arrangement were identified.

## Discussion

The clinical characteristics of the patients studied in this study were similar to those reported in the literature. The predominance of OLP in female patients and in those in the fifth through seventh decades of life was observed in the present study, in agreement with other reports (3,10,11). The buccal mucosa was the most common site of OLP lesions in our sample, which is similar to the findings of previous studies (3,10-12). The red type of OLP [atrophic and erosive lesions] was predominant in this Egyptian sample of patients [59.37% of patients had atrophic OLP meanwhile 20.3% developed erosive type of OLP], which is not in agreement with a study conducted worldwide that found prevalence of the white forms of OLP in 66.9% in a study performed by Cekie-Arambasin *et al.* (13); 88.5% in another study conducted by Persic *et al.* (14) and 62% as documented by Thongprasom *et al.* (15). Similar results were also not observed in an Iranian sample in which 77% white keratotic forms were seen (16). In our sample, 14.06% of patients were HCV serum-positive. Epidemiological data suggest that LP may be associated significantly with HCV infection in various parts of the world with presence of geographical difference. This difference may be due to immunogenetic factors, duration of HCV infection, and differences in study design (17).

The Egyptian Demographic Health Survey [EDHS], a cross sectional survey including hepatitis C virus [HCV] biomarkers, was conducted in 2008 on a large nationally representative sample. It estimated HCV prevalence among the 15–59 years age group to be 14.7%. Anti-HCV circulating antibodies thus appear to be more common in patients with OLP among the general population reflect a national level of epidemic.

Accordingly, Egypt has the highest HCV prevalence in the world (18,19). This is in line with the present study in which HCV infected patients with OLP was representing 14.06 % of the study sample showing the highest prevalence among the patients.

Diabetes mellitus, mainly type 2, affected 15.63% of the OLP patients in our sample, reflecting the prevalence of diabetes [types 1 and 2] in the general population of Egypt [9.3%] (20).

OLP is considered to be a potentially malignant disorder of the oral mucosa (21,22). The most important complication of this disease may be the development of oral squamous cell carcinoma, although this is a very controversial topic (23). In our sample, only two patients [3.13% 2/64 OLP patients] developed squamous cell carcinoma at a site with confirmed OLP lesions. The malignant transformations occurred in one erosive and one atrophic type of lichen planus, with no dysplasia noted in the initial biopsies. This percentage is rather similar to other reported data. A study in south-eastern Spain (12) found a malignization rate of 0.90% [8/550 OLP patients].

Carbone *et al.* (24) reported that 1.85% of 808 OLP patients in Italy developed oral carcinoma during the follow-up period. Thirteen of 690 [1.9%] British OLP patients (10) developed oral squamous carcinoma and four of 141 [2.8%] patients in Switzerland (25) showed oral carcinoma at a site with OLP lesions. A study conducted in Saudi Arabia showed malignant transformation in 5.41% [4/74 OLP patients] which is in accordance with our study (26).

Given that chronic inflammation has been associated causally with various types of cancer, the malignant transformation of OLP may be related to, or dependent on, a series of molecular stimuli originating in the inflammatory infiltrate [e.g. cytokine and chemokine release by infiltrating T cells]. These stimuli may induce fundamental protein changes in oral epithelial cells, leading to the progression of OLP to oral squamous cell carcinoma (27).

In this study, 32% of patients reported an exacerbation of OLP symptoms in periods of greater emotional tension and/or anxiety. In a recent paper, Manolache *et al.* (28) evaluated the possible role of stress in the on-set/extension of cutaneous lichen planus in patients treated at the dermatological department of Cetatea Histria Polyclinic in Bucharest, Romania. In this case-control study, the authors identified at least one potentially stressful situation in 31 cases [67.39%], compared with the occurrence of such situations in 10 control patients [21.73%]. Psychological disturbances have been investigated in the etiopathogenesis of OLP. Stress, as well as other psychological alterations, seems to modify and promote dysregulation of immune functions by altering the Th1/Th2 cytokine balance and increasing Th2 response, which is associated with the development of autoimmune diseases (29) which is in agreement with our study in which OLP occurred in 4.69% following stressful situations in medically free patients. Although some investigations (30) have failed to replicate the association between OLP and stressful events, psychological/ psychiatric services should be combined with conventional therapy for these patients to avoid the occurrence of somatisation and to prevent disease exacerbation. There is no literature data indicating an elevated prevalence of smoking or alcohol consumption among patients with OLP compared to the general population (13), a finding also observed in the present study regarding smoking only. The cheek mucosa was the site most affected, followed by the lips, gingiva and tongue, which is in agreement with other report (9). Extraoral manifestations were observed in 33.67% of the patients studied and exclusive oral lesion in 66.33%. According to the literature, 50% of all patients with lichen planus simultaneously present skin and oral lesions, whereas 25% present only oral lesions. In contrast, other studies have reported cutaneous involvement in less than 17% of patients with OLP. The atrophic form was the most frequent, followed by the erosive form. These two forms were found to be associated or not with other forms, as also reported by other investigator (9,13). Burning sensation was the most frequent symptom and was observed in 60% of cases; of these, 76.08% had the erosive form of OLP. Similar results have been reported in previous studies (5,9,13).

## Conclusions

Although observational retrospective studies have various limitations, to the best of our knowledge, no similar study has been conducted among the Egyptian population. The present study revealed the prevalence of OLP among middle-aged women. Atrophic lesions were most frequent, followed by the erosive form. Anti-HCV circulating antibodies were more common in patients with OLP and, notably, OLP was associated with Diabetes mellitus in 15.63% of patients.
